# Co-occurrence of adult abuse and child abuse: analysis of the phenomenon

**DOI:** 10.5249/jivr.v14i1.1640

**Published:** 2022-01

**Authors:** Marta Kożybska, Marta Giezek, Paulina Zabielska, Barbara Masna, Jacek Ciechowicz, Monika Paszkiewicz, Artur Kotwas, Beata Karakiewicz

**Affiliations:** ^*a*^ Subdepartment of Medical Law, Department of Social Medicine, Pomeranian Medical University in Szczecin, Poland.; ^*b*^ Subdepartment of Social Medicine and Public Health, Department of Social Medicine, Pomeranian Medical University in Szczecin, Poland.; ^*c*^ Centre for People with Special Needs in Szczecin, ul. Tartaczna 14, 70-893 Szczecin, Poland.; ^*d*^ Municipal Family Support Center in Szczecin, ul. Sikorskiego 3, 70-323 Szczecin, Poland.

**Keywords:** Domestic Violence, Child Abuse, Intimate Partner Violence

## Abstract

**Background:**

The purpose of this study was to analyze the co-occurrence of adult and child abuse based on the reports collected from the Polish police and social welfare institutions.

**Methods:**

The study involved data concerning 468 households in Szczecin (Poland) inhabited by children where acts of violence between adults took place. The presented data refer to the years 2012-2103. The data came from so called Blue Card files, i.e. documents issued by the police and social workers in cases of domestic abuse, providing information about its forms, perpetrators, and victims.

**Results:**

Domestic violence usually occurs between spouses and cohabitees (78%). The perpetrator was usually a man (88%). Violence usually lasted from 1 up to 3 years (30.0%). The most common forms of physical abuse against adults and children included pushing (79.5% of adults, 22.4% of children) and hitting (64.7% of adults, 16.6% of children), and psychologically abusive behaviors were mostly insults (91.9% of adults, 27.5% of children) and criticism (79.1% of adults, 21.5% of children). This work has shown that the longer the psychological abuse between adults lasts, the greater probability is that it will also be used against children. Child abuse is also associated with putting up resistance to the police by perpetrators.

**Conclusions:**

Summing up, in households where violence between adults is observed, actions should be taken to prevent violence against children.

## Introduction

Violence is one of the oldest social problems that has not been successfully solved yet. It is estimated that 12% of children in Europe are affected by violence; 64% in Asia; 56% in North America; 50% in Africa; and 34% in Latin America.^1^ However, it should be kept in mind that violence is a phenomenon that is difficult to estimate. The prevalence of violence against children varies depending on the form of violence (sexual, physical, emotional, neglect), place of occurrence and gender of the child. The difficulty is the lack of a uniform methodology for collecting this type of information. On the one hand, some researchers obtain this data using the self-reported method, and on the other hand, data collected by specialized institutions are also presented. This causes significant discrepancies in the obtained results. The literature indicates that in Europe the most common form of violence against children is neglect (27.0% median prevalence rates for both genders), emotional / psychological abuse (21.7% median prevalence rates for both genders), sexual abuse (13.2% median prevalence rates for both genders) and physical violence (12.2% median prevalence rates for both genders).^2^ The World Health Organization's Global Status Report on Preventing Violence Against Children 2020 indicated that violence has long-lasting emotional, social and economic consequences.^3^ It is noted in the literature that its consequences are not limited to an individual who experiences violence, but include entire families, communities and nations, from generation to generation.^1^

An important problem associated with domestic violence is that children are exposed to it. Exposure to violence is usually defined as "being within sight or sound of violence".^4^ Exposure to violence includes child abuse, school violence, and community or neighborhood violence.^5^ Children may also be exposed to intimate partner violence (IPV). This phenomenon is defined as an intimate partner’s behavior that causes physical, sexual or psychological harm, including acts of physical aggression, sexual coercion, psychological abuse and controlling behaviors. The term "intimate partner" includes the following people: a husband, cohabiting partner, boyfriend or lover, or ex-husband, ex-partner, ex-boyfriend or ex-lover.^6^ In the past, child exposure to IPV was referred to as IPV "witnessing". However, it is nowadays emphasized that this exposure occurs not only when the child sees or hears violence directly, but always when they are aware of it.^7^ Thus, a Canadian Incidence Study of Reported Child Abuse and Neglect distinguishes three forms of IPV exposure: indirect exposure to physical violence (the child overheard violence but did not see the effects of violence such as a direct injury, or the child was informed about violence by someone or overheard a conversation about violence), direct exposure to physical violence (the child is a direct witness of violence), and exposure to emotional violence (the child is directly or indirectly exposed to emotional violence between partners).^8,9^ The frequency of IPV is significant.^4,6^ The World Health Organization (WHO) estimates that 30% of women around the world have experienced IPV.^6^ This means that the number of children who witness violence is huge. Individual studies point to different prevalence of IPV exposure, the percentage of children who witness IPV is indicated as 8-25% in high-income countries and 10-39% in middle income countries.^10,11^ This problem is of particular importance during the COVID-19 pandemic.^12^ Children living in homes where there is violence between partners are more likely to experience maltreatment^13,14^ and many other adverse consequences, for example, depression.^15^ Research indicates that adverse childhood experiences, which also includes IPV, may even result in the development of cancer.^16^ Both child exposure to IPV and child maltreatment are described as the "double whammy effect" in the literature.^17^

**Prevention of violence**

One of the tools for preventing violence against children is legal norms. In 1989, the United Nations ratified the Convention on the Rights of the Child, recognizing freedom from violence as children’s fundamental right. Also, the contemporary law-making activities of the United Nations take into account the issue of violence against children. The 2030 Agenda for Sustainable Development aims to totally eliminate violence against the child.^1,18^

The Blue Card procedure has been introduced to the Polish law in order to counteract domestic violence. It can be initiated in cases of suspected domestic violence by the police, social workers, school psychologists, or healthcare workers either on their own initiative or on the victim’s request. The initiation of the Blue Card procedure causes that information about the suspicion of domestic abuse is passed to social support organizations, such as the police, as well as social welfare, healthcare, and educational institutions. Representatives of these institutions meet and draw up an appropriate family assistance program.^19,20^

The Blue Card is a document containing information about perpetrators and victims of abuse (name, surname, kinship, age), children living in the same place (age), forms and duration of adult and child abuse, the behavior of perpetrators during the initiation of the Blue Card procedure, and witnesses of abuse (name, surname, kinship with the victim). Additionally, the Blue Card includes information about offenders that can be useful while developing the plan of assistance. This information is obtained by asking questions whether the offender has already been punished for violent crime or potentially violent crime; abuses alcohol, psychoactive substances or drugs; has been receiving psychiatric treatment; has any weapons. The next part of the questionnaire concerns interventions undertaken due to abusive behavior so far (e.g. direct coercion, temporary detention, detention in a detoxification center).

**Factors associated with the use of violence analyzed in the study**

In this study we analyzed a relationship between the co-occurrence of adult and child abuse and such factors as: the kinship of perpetrators and victims, duration of abuse, perpetrator’s criminal record, as well as narcotic, psychotropic and medical drug abuse, history of psychiatric treatment, possession of weapons, behavior at the time of establishing the Blue Card. The presented data refer to domestic violence incidents in the city of Szczecin (Poland) in the years 2012-2013. They do not show lifetime prevalence.

Analysis of the degree of the kinship between perpetrators and victims will enable establishing if only intimate partner violence (IPV) or also violence between other individuals than partners exposes a child to abuse. It can be, for example, violence between grandparents, uncles, adult sisters and brothers, and parents. We also took into account the behavior of the perpetrator at moment of establishing the Blue Card (is he/she aggressive or calm). Establishing the duration of violence will provide the answer to the question if there is a relationship between long-lasting violence between adults and abusive behaviors against children. The literature shows that variables such as criminal record,^21-23^ alcohol and drug abuse,^21-25^ psychiatric treatment^21,22^ are related to domestic abuse and abuse against a child. This study is to verify whether these factors entail using violence against children at homes in which violence originally occurred only between adults.

Effective prevention of violence should be based on reliable research outcomes concerning the risk factors for domestic violence. The purpose of this study was to analyze the co-occurrence of adult and child abuse based on the Polish violence prevention procedures. This paper enriches the current state of knowledge by identifying the risk factors for violence against children in households where the main problem of police and social assistance interventions is violence between adults. 

## Methods 

The research method was the analysis of the Blue Card documentation. All Blue Cards are sent to social welfare institutions, where they are kept. We obtained access to the Blue Cards with the consent of the Bioethics Committee, and the director of the Municipal Family Support Center in Szczecin after training on the protection of personal data. 

The present study analyzed all BCs from years 2012-2013 in one of the largest metropolitan communities in Poland (the city of Szczecin; 402,067 inhabitants, 170,300 households).^26,27^ There was a total of 1299 reports of violence, 576 in 2012 and 723 in 2013. A further analysis included households with violence between adults in which a cohabiting person was aged 0-18 years. We found 468 of such households. In all those cases the main cause of police/social interventions was violence between adults. Table 1 shows information included in the Blue Cards which were analyzed in this study. The presented results concern the city of Szczecinv (Poland) and include information collected by authorized officers during the establishment of the Blue Cards. These data refer to the description of violence at the time the Blue Card was established (2012-2013), and not to the occurrence of violence during the lifetime. 

**Table 1:** Information included in the Blue Cards taken into account in the research.

Victim:	
	• sex
	• kinship with the perpetrator
	• forms of abuse
	• duration of abuse
Children living in the same household:	
	• age
	• is abuse used against children
	• forms of abuse against children
Offender:	
	• sex
	• if was punished for the crime of violence or threat of its use
	• if abuses alcohol, narcotic, psychotropic or medical drugs
	• if was treated psychiatrically
	• if owns a gun
	• alcohol content level in offender’s blood
	• offender’s behavior during the initiation of the Blue Card procedure:
	o difficulty in establishing contact
	o calm
	o tearful
	o frightened
	o avoids conversation
	o aggressive
	o resists the police
Are there visible traces of fight at home	

Statistical inference was based on Pearson’s chi-square independence test. The level of significance was set at α = 0.05. Additionally, we used multiple and bivariate logistic regression to determine the influence of explanatory variables on the odds ratio (OR) of physical and psychological abuse against adults and children. Statistical analysis was performed using SPSS software. 

## Results

**Description of the studied group**

**Figure 1:**
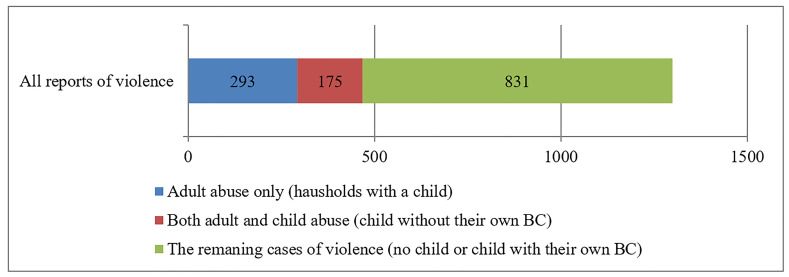
Abuse subgroups

Of the full sample of 1299 reports of violence 468 (36.0%) concerned violence between adults in households with a child. In those 468 households 293 (62.6%) cases of violence against adult only were found while 175 (37.4%) against both the adult and the child were noted (see Figure 1).

The extracted 468 households were inhabited by a total of 785 children. The majority of the children were 10 years old or younger. A great majority of the adult respondents experiencing violence were women and the perpetrators were mainly men (Table 2 ).

**Table 2:** Descriptive data of the study sample.

		n	%
Number of households where abuse occurred between adults living with children		468	100.0
Victim’s sex			
	women	426	91.0
	men	42	9.0
Perpetrator’s sex			
	women	54	11.5
	men	413	88.3
Number of children in the households		785	-
Children’s age			
	0-10 years	461	58.7*
	11-18 years	324	41.2*
Number of households with co-occurring child abuse		175	37.4
Presence of traces of fight in the household		88	18.8

*a total number of children (785) was regarded as 100%

**Characteristics of violence in the examined households**

The most common forms of physical violence in the group of children and adults were cushing, hitting, slapping, bruises. The most common forms of psychological violence included insults, criticism, humilitation (see Table 3 ). A part of characteristics shown in Table 4 connected with the violent person and the types of violence was statistically significant. It was: relatedness between the violent person and the person experiencing violence, the duration of violence, the violent persons’ alcohol abuse, psychiatric treatment of a violent person, the violent person’s behavior at the time of establishing the Blue Card, the forms of violence.

**Table 3:** Forms of Violence Used in Households with Children.

	Adult (N = 468)		Child (N = 175)	
	n	%	n	%
**Physicalviolence**				
pushing	372	79.5%	101	22.4%
hitting	303	64.7%	75	16.6%
slapping	211	45.1%	39	8.7%
bruises	208	44.4%	39	8.7%
twisting	163	34.8%	24	5.3%
choking	109	23.3%	18	4.0%
scratches	149	31.8%	23	5.1%
kicking	141	30.1%	25	5.5%
bleeding	83	17.7%	6	1.3%
burns	3	0.6%	2	0.4%
**Psychological violence**				
insults	430	91.9%	124	27.5%
criticism	370	79.1%	97	21.5%
humiliation	366	78.2%	90	20.0%
threats	338	72.2%	73	16.2%
ridicule	325	69.4%	83	18.4%
regular troubling	309	66.0%	85	18.9%
control	207	44.2%	54	12.0%
limiting contacts	166	35.5%	40	8.9%
isolation	132	28.2%	30	6.7%
demoralization	82	17.5%	65	14.4%

**Table 4:** Selected characteristics of the phenomenon of violence in households with children.

	TOTAL N = 468%	Psychological violence against adult n = 442		Physical violence against adult n = 393		Psychological violence against child n = 159		Physical violence against child n = 119	
		Row %	Chi^2^, p-value	Row %	Chi^2^, p-value	Row %	Chi^2^, p-value	Row %	Chi^2^, p-value
Age the violent person									
18-40	45.1	44.1	0.198	47.3	0.084	44.1	0.256	46.2	0.613
41-60	33.1	33.9	0.124	31.3	0.061	34.6	0.256	30.3	0.596
61-80	3.0	3.2		3.3		1.3		1.7	
lack of data	18.8	18.8		18.1		20.0		21.8	
Degree of relatedness between the violent person and the person experiencing violence									
spouse	51.3	54.1		55.0		56.0		51.3	
no relationship (e.g. cohabitee)	26.9	26.7		27.0		24.5		23.5	
ascendant	9.4	6.8	71.398	6.1	37.197	10.1	9.042	16.0	10.347
descendant	5.8	5.9	0.000**	5.3	0.000**	3.8	0.107	4.2	0.066
collateral	3.2	3.2		2.8		3.8		1.7	
stranger (e.g. neighbor)	0.9	0.9		1.0		0.6		0.8	
Duration of violence									
1-4 weeks	4.3	3.8		3.6		3.1		2.5	
2-12 months	28.2	28.3	9.865	28.5	4.761	25.8	11.708	21.0	10.689
1-3 years	29.7	30.3	0.043**	30.0	0.313	28.9	0.020**	27.7	0.030**
4-7 years	15.4	16.3		16.3		20.1		20.2	
longer	12.2	12.4		12.7		13.8		12.6	
Violent person was punished for the crime of violence or threat of its use	12.2	12.2	0.002	12.7	0.612	11.9	0.144	12.6	1.163
			0.962		0.434		0.705		0.281
Violent person abuses alcohol	67.5	69.5	8.192	69.7	2.594	78.0	6.567	69.7	0.456
			0.004**		0.107		0.010**		0.500
Violent person abuses narcotic, psychotropic or medical drugs	10.9	11.3	0.841	11.5	0.638	11.9	0.831	10.1	0.015
			0.359		0.425		0.362		0.901
Violent person was treated psychiatrically	10.0	10.2	0.013	9.9	0.059	13.8	3.267	13.4	2.659
			0.910		0.808		0.071**		0.103
Violent person owns a gun	1.7	1.8	0.280	1.5	0.675	2.5	0.707	1.7	0.667
			0.597		0.411		0.400		0.414
Alcohol content level in violent person’s blood									
0.2-1.0	1.9	2.0		2.3		2.5		2.5	
1.1-2.0	12.0	12.0	1.253	12.7	1.679	17.0	0.070	16.0	0.053
2.1-3.0	17.5	17.2	0.263	17.8	0.195	15.1	0.791	16.8	0.817
3.1-4.0	3.4	3.4		4.1		1.9		2.5	
Violent person’s behavior: difficulty in establishing contacts	18.8	19.2	0.320	19.1	0.061	24.5	20.032	21.0	1.470
			0.572		0.805		0.000**		0.225
Violent person’s behavior: calm	20.3	19.7	4.159	20.4	0.539	17.6	2.496	23.5	0.234
			0.041**		0.463		0.114		0.629
Violent person’s behavior: tearful	7.9	7.2	8.085	7.1	4.276	6.3	1.782	5.9	2.399
			0.004**		0.039**		0.182		0.121
Violent person’s behavior: frightened	0.9	0.7	3.937	1.0	0.618	1.3	0.091	2.5	3.187
			0.047**		0.432		0.763		0.074**
Violent person’s behavior: avoids conversation	14.5	15.2	2.237	14.5	0.315	21.4	14.151	21.8	6.835
			0.135		0.574		0.000**		0.009**
Violent person’s behavior: aggressive	34.0	35.7	12.726	36.1	3.201	38.4	3.180	36.1	0.042
			0.000**		0.074**		0.075**		0.839
Violent person’s behavior: resists the police	12.4	13.1	3.315	12.5	0.257	20.1	9.310	16.8	0.846
			0.069**		0.612		0.002**		0.358
Psychological violence against child	34.0	33.5	0.020	32.1	2.692	-	-	-	-
			0.889		0.101				
Physical violence against child	25.4	22.9	12.457	25.2	0.049	-	-	-	-
			0.000**		0.824				
Psychological violence against adult	94.4	-	-	-	-	93.1	0.020	84.9	12.457
							0.889		0.000**
Physical violence against adult	84.0	-	-	-	-	79.2	2.692	83.2	0.049
							0.101		0.824

The data do not ad dup to 100% due to gaps in BCs. ** p< .05 *** .091 >p> .051

**The risk factors for violence**

The next step was to determine which characteristics affect the prevalence of different forms of violence against the child and against the adult. The factors that increase the odds of psychological abuse against an adult were: prolonged duration of violence (OR=1.73), violent persons’ alcohol abuse (OR=3.50), aggressive behavior of a violent person (OR=16.71). Aggressive behavior of a violent person was positively associated with physical violence (OR=1.87). The factors that increase the odds of psychological abuse against a child were prolonged duration of violence (OR=1.37), violent persons’ alcohol abuse (OR=2.10), difficulty in establishing contact with the violent person(OR=6.57), avoidance of conversation by a violent person (OR=5.03), resisting the police (OR=3.49). The odds of physical violence against a child were increased by: prolonged duration of violence (OR=1.35) and avoidance of conversation by a violent person (OR=5.03) (Table 5 ).

**Table 5:** Bivariate logistic regression of forms of violence and selected variables.

	Psychological violence against adult OR (95% CI)	Physical violence against adult OR (95% CI)	Psychological violence against child OR (95% CI)	Physical violence against child OR (95% CI)
Duration of violence	1.73 (1.04-2.89) **	1.21 (0.94-1.56)	1.37 (1.07-1.74) **	1.35 (1.06-1.72) **
Violent person was punished for the crime of violence or threat of its use	1.03 (0.29-3.71)	0.71 (0.30-1.67)	0.85 (0.37-1.95)	0.63 (0.28-1.45)
Violent person abuses alcohol	3.50 (1.41-8.69) **	1.58 (0.90-2.76)	2.10 (1.18-3.72) **	1.21 (0.68-2.16)
Violent person abuses narcotic, psychotropic or medical drugs	2.52 (0.33-19.64)	1.44 (0.58-3.57)	1.46 (0.64-3.33)	1.05 (0.46-2.41)
Violent person was treated psychiatrically	0.91 (0.19-4.22)	0.9 (0.39-2.05)	2.26 (0.91-5.58) *	1.97 (0.86-4.51)
Violent person owns a gun	56606936 (0-.)	0.51 (0.10-2.60)	2.50 (0.27-22.74)	2.63 (0.23-29.53)
Violent person’s behavior: difficulty in establishing contacts	1.46 (0.38-5.55)	0.91 (0.44-1.88)	6.57 (2.70-15.98) ***	1.54 (0.76-3.11)
Violent person’s behavior: calm	0.32 (0.10-1.00) *	0.77 (0.37-1.56)	0.58 (0.30-1.13)	1.17 (0.60-2.29)
Violent person’s behavior: tearful	0.20 (0.06-0.67) **	0.42 (0.17-0.97) **	0.54 (0.21-1.34)	0.47 (0.17-1.24)
Violent person’s behavior: frightened	0.13 (0.01-1.37) *	250184160 (0-.)	1.44 (0.12-16.32)	1753944138 (0-.)
Violent person’s behavior: avoids conversation	4.25 (0.54-33.34)	0.8 (0.37-1.72)	5.03 (2.06-12.28) ***	2.72 (1.26-5.85) **
Violent person’s behavior: aggressive	16.71 (2.12-131.38) **	1.87 (0.93-3.72) **	1.79 (0.94-3.42) *	0.93 (0.48-1.78)
Violent person’s behavior: resists the police	93527492 (0-.)	0.81 (0.35-1.83)	3.49 (1.52-8.00) **	1.42 (0.67-3.01)
Alcohol content level in violent person’s blood	0.73 (0.29-1.78)	0.95 (0.48-1.86)	0.65 (0.36-1.15)	0.87 (0.49-1.51)
Age the violent person	2.19 (0.94-5.10) *	0.68 (0.42-1.07)	0.82 (0.52-1.30)	0.79 (0.50-1.26)

* p < .1, ** p < .05 , *** p < 0,001

The results of multivariate analysis only confirmed the connection between duration of violence and psychological abuse against children in the households where psychological abuse against adults occurred, and between resisting by perpetrators to the police and both psychological and physical abuse against children in the households with physical and psychological abuse observed between adults (Table 6). 

**Table 6:** Multivariate analysis of risk factors of child abuse.

	Psychological violence against child when psychological violence against adult	Physical violence against child when physical violence against adult	Risk factors of child abuse (physical and psychological)
	OR (95% CI)	OR (95% CI)	OR (95% CI)
Duration of violence	1.666 (1.006-2.758) *	1.286 (0.701-2.359)	1.435 (0.901-2.286)
Violent person was punished for the crime of violence or threat of its use	4.131 (0.458-37.224)	1.117 (0.126-9.891)	4.934 (0.607-40.13)
Violent person abuses alcohol	0.687 (0.224-2.104)	1.546 (0.423-5.647)	0.915 (0.32-2.619)
Violent person abuses narcotic, psychotropic or medical drugs	0.75 (0.067-8.385)	0.337 (0.018-6.353)	0.897 (0.127-6.343)
Violent person was treated psychiatrically	3.853 (0.471-31.525)	1.585 (0.099-25.46)	4.917 (0.675-35.822)
Violent person owns a gun	0 (0-.)	20538348.346 (0-.)	0 (0-.)
Violent person’s behavior: difficulty in establishing contacts	2.457 (0.572-10.551)	1.455 (0.222-9.541)	2.106 (0.582-7.616)
Violent person’s behavior: calm	0.55 (0.122-2.474)	2.257 (0.289-17.596)	0.908 (0.225-3.67)
Violent person’s behavior: tearful	0.778 (0.137-4.407)	0 (0-.)	0.591 (0.124-2.815)
Violent person’s behavior: frightened	2.714 (0.061-120.61)	233014280.294 (0-.)	2.544 (0.075-86.406)
Violent person’s behavior: avoids conversation	0.564 (0.139-2.283)	1.171 (0.248-5.523)	0.476 (0.124-1.828)
Violent person’s behavior: aggressive	0.373 (0.074-1.89)	0.577 (0.058-5.725)	0.444 (0.103-1.923)
Violent person’s behavior: resists the police	9.068 (1.977-41.585) *	7.824 (1.22-50.191) *	9.448 (2.077-42.984) *
Age the violent person	0.76 (0.315-1.835)	0.923 (0.326-2.614)	0.893 (0.395-2.019)

## Discussion

The authors’ research focused on a widespread exposure of children to domestic violence, which is violence that occurs not only between parents or parents and partners but also between other cohabitees. Moreover, the study attempted to fill the gap in the current research, indicating the forms of violence children are exposed to and by pointing to the forms which children experience directly. 

The authors’ research has shown that domestic violence in households with children is the most frequent between spouses and cohabitees. This concerned both physical and psychological violence. In the study on children's exposure to violence, the phenomenon of IPV is very important, because IPV increases the risk of child abuse.^15,28^

Contrary to Tajima’s^29^ results it is worth noticing that co-occurrence of violence against adult and children is a common phenomenon and violence against adult was often accompanied by child abuse. This data provides some insight into the scale of the phenomenon of violence in society, but little is known about the forms of violence which children are exposed to,^4^ and the risk factors for violence. The literature indicates that there is still insufficient knowledge on the types of IPV-related violence that children are exposed to.^4,30^ The authors’ research fills this gap. The outcomes clearly show that in households with children, the most common forms of physical violence between adults were pushing (79.5%), hitting (64.7%) and slapping (45.1%). The most frequent forms of psychological violence were insults (91.9%), criticism (79.1%), and humiliation (78.2%). To date, it has been shown that there is a relationship between physical violence against children and the prevalence of domestic violence.^22,31^ We have supplemented this knowledge by verifying if this relationship depends on physical or psychological violence. It has been shown that the use of physical violence against the child is significantly related to using psychological violence between adults. 

We also focused on factors associated with using violence against children. The previous research has identified factors such as loss of job by parents,^32^ poverty,^33^ criminality,^34^ mental illness, substance abuse, incarceration of a household member, violence against the mother, separation of parents.^35^ During the COVID-19 pandemic, there is evidence of a greater prevalence of violence against children as a result of isolation and social distance,^2^ but not all studies confirm this.^36^

In the discussion on the risk factors for child abuse, the IPV risk factors cannot be ignored. Among these factors the following have been distinguished so far: low level of education (both men and women), violence against women in childhood, low socio-economic status, rural place of residence, higher number of children and separate or divorced marital status.^24^ The partner’s unemployment was a factor as well,^37,38^ although Indian research does not support this thesis.^24^ The co-occurrence of domestic violence with physical violence against children correlates with such factors as lower education, poor health and depression in the family.^22,23^

The authors’ research has identified such factors as: behavior of a person who uses violence, psychological violence as the risk factor for physical violence, alcohol abuse, duration of violence, mental illness. As far as the behavior of a person using domestic violence is concerned, it has been shown that women's exposure to severe physical injuries is related to jealousy, suspicion and control from their partners.^24^ The research revealed that perpetrator’s anxiety and aggression were associated with violence between adults and resistance to the police has with child abuse. There was no statistically significant relationship between prior conviction of the perpetrator for using violence or a threat of its use and the use of a given form of violence. However, it should be emphasized that in up to 12.2% of the analyzed households, the perpetrator of violence had a criminal history record. The literature confirms the relationship between crime and domestic violence^39^ and the use of child maltreatment.^21^ It has also been proven that co-occurrence of domestic violence and physical violence against the child is related to arresting the father for crime rather than for domestic violence.^22,23^

In the presented research, the relation has been shown between the use of psychological violence between adults and the use of physical violence against a child. Sabri et al. point out that psychological abuse is a risk factor for severe physical abuse between adults^24^ however, the authors’ research did not confirm this thesis. 

An important risk factor for violence is alcohol use. It correlates with the prevalence of violence between partners ^24,25^ and child maltreatment.^21^ The co-occurrence of domestic violence and physical violence against the child is related to both alcohol and drug use.^22,23,29^ In our research, the fact of alcohol abuse by the perpetrator was associated with psychological violence against the adult. Multivariate analysis did not confirm it to be related to child abuse. It was not found that the perpetrator's psychiatric illness correlated with physical violence against the child or against the adult. Nevertheless, other research suggests that a psychiatric illness can be a factor associated with child abuse and exposure to domestic violence.^21,22^ Gonzalez et al. reported that a caregiver’s mental illness is a significant risk factor for child behavior problems related to IPV exposure.^7^

For designing the preventive measures, an important finding from the authors’ research is that a long-term prevalence of violence between adults also correlated with psychological and physical violence against the child. This justifies the need for early interventions in environments where domestic violence occurs. Moreover, Sabri et al. have demonstrated a relationship according to which being in a long-term relationship is related to experiencing a serious trauma as a result of IPV.^24^

To sum up, the fact that children are witnesses of violence is a major social problem. Violence in households with children lasts for a long time and is reported late. The fact that there is just violence between adults does not mean that the child is not at risk. The results obtained in this study suggest that the longer violence between adults maintains, the higher the risk that the child will not only be an indirect or direct witness, but that violence will also be directed against him or her. It has also been shown that in households where the adult is affected by psychological violence, the child is at risk of physical violence. 

Thus, the results of the research justify early interventions to eliminate domestic violence. Early interventions, also when there is only psychological violence between partners, taken especially against the perpetrators who refuse to cooperate, can help to protect children against experiencing domestic violence directly.

**Limitations**

This study had a number of limitations. It should be noted that this research did not directly assess children’s exposure to adult domestic violence. Nevertheless, the literature indicates that one of the forms of exposure to domestic violence is being ostensibly unaware, that is, situations when the child does not know about violence between parents.^40^ Among the analyzed risk factors there were no such factors as family's financial situation, overcrowding of the household, or education of adult inhabitants. However, analysis of these aspects was not possible because such information was not included in the Blue Cards, which were the research material of this study.

**Acknowledgement**

The authors would like to thank the employees of the Municipal Family Support Center in Szczecin for providing access to blue cards. 
